# Prevalence and Characterization of Methicillin-Resistant *Staphylococcus aureus* from Community- and Hospital-Associated Infections: A Tertiary Care Center Study

**DOI:** 10.3390/antibiotics10020197

**Published:** 2021-02-18

**Authors:** Puthiya Purayil Preeja, Sanath H. Kumar, Veena Shetty

**Affiliations:** 1 Department of Microbiology, KS Hegde Medical Academy, Nitte (Deemed to Be University), Mangalore 5750181, India; preejasharath@gmail.com; 2 QC Laboratory, Post Harvest Technology, ICAR-Central Institute of Fisheries Education, Mumbai 400061, India; sanathkumar@cife.edu.in

**Keywords:** CA-MRSA, *PVL*, SCC*mec* typing, multi-drug resistance, HA-MRSA

## Abstract

The community-associated methicillin-resistant *Staphylococcus aureus* (CA-MRSA) has become increasingly prevalent in both community and hospital settings. The aim of this study was to determine the prevalence, molecular characteristics and antibiotic resistance profiles of CA-MRSA from community- and hospital-associated infections in a tertiary care hospital in Mangalore, India. Of 520 *S. aureus* isolates, 362 were from inpatients (IP) and 158 were from outpatients (OP). One-hundred and thirty-two MRSA isolates obtained from 94 inpatients and 38 outpatients with complete clinical details were further analyzed. Of these, 81 (61.4%) were CA-MRSA (IP-47.9%, OP-94.7%) and 51 (38.6%) were HA-MRSA (IP-52.1%, OP-5.3%). All (100%) MRSA isolates were *mecA* gene positive. SCC*mec* typing identified SCC*mec* type IV (50.6%) and SCC*mec* type V (66.7%) in CA-MRSA, while SCC*mec* type I (41.2%), SCC*mec* type III (19.6%), SCC*mec* type IV (31.4%) and SCC*mec* type V (25.5%) were detected in HA-MRSA isolates. The Panton–Valentine Leukocidin (PVL) gene was found in 70.4% of CA-MRSA, 43.1% of HA-MRSA with SCC*mec* type IV and SCC*mec* type V, and in 7.8% of true HA-MRSA. The antibiotic resistance profiles were determined by the disc diffusion method. Resistance to cefoxitin was used to identify MRSA. A significant difference *(p* < 0.05) was observed between CA-MRSA and HA-MRSA with respect to resistance against cephalexin, cefotaxime, levofloxacin, linezolid and teicoplanin. CA-MRSA was predominantly resistant to ciprofloxacin (86.4%), erythromycin (66.7%), ofloxacin (49.4%), cefotaxime (44.4%), gentamicin (40.7%) and clindamycin (40.7%), while HA-MRSA showed resistance against ciprofloxacin (80.4%), erythromycin (80.1%), cefotaxime (70.6%),ofloxacin (58.8%), clindamycin (47.1%) and levofloxacin (41.2%).This study reports the prevalence of CA-MRSA in community and hospital settings and the possibility of multidrug-resistant CA-MRSA replacing HA-MRSA in hospitals. The observations from our study emphasize the need for urgent measures to manage this emerging crisis in healthcare settings.

## 1. Introduction

*Staphylococcus aureus* is a highly versatile bacterial pathogen capable of causing a wide range of infections in humans, from mild skin infections to severe systemic diseases such as pneumonia. Since the first report of methicillin-resistant *S. aureus* (MRSA) in the 1960s, MRSA has been recognized as a pathogen of global concern [1]. Although MRSA infections were originally acquired only from hospital settings (HA-MRSA), community outbreaks were first reported in the 1990s from Australia and the United States of America, and subsequently from across the world [2,3]. Community-associated methicillin-resistant *S. aureus* (CA-MRSA) strains were originally restricted to the community and found mainly in healthy, young patients [4]. However, CA-MRSA infections are now being increasingly reported in community and hospital settings as well [5,6,7]. According to the US Centers for Disease Control and Prevention (CDC), a MRSA infection can be categorized as CA-MRSA when the patient has no history of surgery, hospitalization or residence in a long-term care facility within the year before infection, has no percutaneous device or indwelling catheter, has not undergone dialysis within the previous year, hospitalization <48 h before MRSA culture, or has no history of previous MRSA infection or colonization [8]. CA-MRSA can cause infections in healthy individuals with no hospital-associated risk factors, is more susceptible to non-β-lactam antibiotics and possesses the virulence-associated Panton–Valentine Leukocidin (PVL) gene, which is responsible for leucocytosis and tissue necrosis [9,10]. Methicillin resistance, due to altered penicillin binding protein (PBP-2a), is encoded by the *mecA* gene present on a mobile genetic element present in staphylococcal cassette chromosome *mec*(SCC*mec*) [11]. So far, 14 SCC*mec* sequence types have been reported [12]. Studies showed that CA-MRSA contain SCC*mec* types IV and V, mostly with the PVL gene, while HA-MRSA carry SCC*mec* types I, II and III [13]. HA-MRSA are commonly associated with nosocomial infections and are resistant to non-β-lactam antibiotics such as fluoroquinolones, macrolides and aminoglycosides [14]. HA-MRSA generally do not possess the PVL gene and belong to the SCC*mec* types I, II and III [15]. CA-MRSA are often resistant to multiple drugs including β-lactams, aminoglycosides, fluoroquinolones and tetracyclines [16,17,18]. The infiltration of CA-MRSA into hospital settings in different countries is a major concern [19,20,21,22]. CA-MRSA infections in Asian countries range from 2.5% to 39% [23]. The occurrence of multidrug-resistant PVL-positive CA-MRSA in hospital settings is frequently reported in India [24,25].

The literature shows that limited data is available on the molecular epidemiology, prevalence and antibiogram of CA-MRSA from the southern part of India. The aim of this study was to gain a better understanding of the prevalence, molecular characteristics and antibiotic resistance profiles of CA-MRSA from community- and hospital-acquired infections in a tertiary care hospital in Mangalore, India. Our study revealed the prevalence of PVL-positive antibiotic-resistant CA-MRSA not only in the community, but also in hospital settings.

## 2. Materials and Methods

This is a cross-sectional study carried out in a tertiary care hospital in Mangalore, India. Ethical approval (NU/CEC/Ph.D- 65/2012) for the study was obtained from the Central Ethical Committee, Nitte (Deemed to be University). The study was carried out from October 2014 to December 2016.

### 2.1. Isolation of S. aureus and Phenotypic Confirmation of MRSA

#### 2.1.1. Isolation of *S. aureus*

The study’s information was given to the patients, and with their consent, the demography, age, sex and other clinical details were documented. The term “outpatient” was used for patients visiting the hospital without a hospital stay, while “inpatient” was used for patients requiring at least an overnight hospitalization. Out of 520 patients diagnosed as having *S. aureus* infection, 132 MRSA isolates with complete clinical details were considered for further study. These 132 MRSAs were isolated from various clinical samples such as pus (111), blood (18), a throat swab (1), body fluids (1) and urine (1) (Table 1).

Blood agar and MacConkey agar (Hi-Media, Mumbai, India) were inoculated with the sample and incubated at 37 °C for 24–48 h. The isolates were identified by colony morphology, Gram staining, catalase, coagulase and mannitol fermentation tests [26]. *S. aureus* ATCC 25923 was used as the reference strain for the biochemical tests.

#### 2.1.2. Phenotypic Confirmation of Methicillin Resistance

Methicillin resistance was tested using a cefoxitin disc (30 µg) by the disc diffusion method on Mueller Hinton agar (Hi-Media, Mumbai). Isolates showing a zone of inhibition of ≤21 mm with cefoxitin were considered methicillin-resistant [27]. Further confirmation of methicillin resistance was done by determining the minimum inhibitory concentration (MIC) using the EzyMIC^TM^ strip (Hi-Media, Mumbai, India). Isolates with a cefoxitin MIC of ≥4 µg/mL were confirmed as MRSA. *S. aureus* ATCC 29213 (methicillin-sensitive, MSSA) and ATCC 43300 (methicillin-resistant, MRSA) were used as the reference strains. CA-MRSA was classified according to the CDC definition [28,29].

#### 2.1.3. Antibiotic Susceptibility Testing

The Kirby–Bauer disc diffusion method was used to determine the antibiotic susceptibility profiles of isolates on Mueller–Hinton agar. The zones of inhibition were interpreted according to the Clinical & Laboratory Standards Institute (CLSI) guidelines [30]. The antibiotics (Hi-Media, Mumbai, India) used were amikacin (30 µg),ampicillin (10 µg), cephalexin (30 µg), cefoxitin (30 µg), cefotaxime (30 µg), ciprofloxacin (5 µg), clindamycin (2 µg), chloramphenicol (30 µg), trimethoprim-sulphamethoxazole (25 µg), doxycycline (30 µg), erythromycin (15 µg), gentamicin (10 µg), levofloxacin (5 µg), linezolid (30 µg), netilmicin (30 µg), oxacillin (1 µg), ofloxacin (5 µg), penicillin (10 U), rifampicin (5 µg), tetracycline (30 µg), tigecycline (15 µg), teicoplanin (30 µg) and vancomycin (30 µg).

### 2.2. Molecular Characterization of MRSA

Methicillin resistance was confirmed by detecting the presence of the *mecA* gene by the polymerase chain reaction (PCR). CA-MRSA and HA-MRSA were differentiated using primers detecting the SCC*mec* and *lukS*/*F-PV* genes, which encode the PVL S/F bicomponent proteins using previously described PCR protocols (Table S1) [31,32,33]. For the preparation of DNA for PCR, a single bacterial colony from the nutrient agar was grown in Luria Bertani (LB) broth at 37 °C for 24 h, and 50 µl of the broth culture was mixed with 450 µL of 1X TE (10 mM Tris pH 8, 1 mM EDTA) buffer. The mixture was heated in a dry bath at 98 °C for 10 min, chilled immediately on ice and centrifuged. The supernatant containing DNA was stored at –20 °C until use.

All amplifications were done in a 30-µL volume consisting of a 10X *Taq* buffer (100 mM Tris–HCl, 500 mM KCl and 15 mM MgCl_2_ (Hi-Media, Mumbai, India)), 200 µM concentrations of each of the four dNTPs, 30 picomoles of forward and reverse primers, 1.5 U of *Taq* polymerase (Hi-Media, Mumbai, India) and 2 µL of DNA template. The amplifications were carried out in a thermocycler (Bio-Rad Laboratories, Inc., USA). The products of the PCR were separated on a 1.5% agarose gel stained with ethidium bromide (0.5 µg/mL) along with a 100 bp DNA ladder (Hi- Media, Mumbai, India) and were photographed using a gel documentation system (Bio-Rad, USA).

The SCC*mec* PCR products were purified using a PCR purification kit (Thermo Fisher Scientific, USA) and sequenced by Sanger’s dideoxy chain termination method (Bioserve Biotechnologies Pvt Ltd., Hyderabad, India).

### 2.3. Statistical Analysis

The data were analyzed using SPSS (Version 20.0, IBM Corp., Armonk, NY, USA) software. The collected information was summarized by using the frequency, percentage, mean and standard deviation. To compare the difference in antibiotic sensitivity with respect to CA-MRSA and HA-MRSA, Chi-square and Fishers exact tests were used.

### 2.4. Nucleotide Sequence Accession Number

The nucleotide sequences derived in this study have been assigned the GenBank accession numbers MK995142 to MK995148 and MK975991 to MK975993.

## 3. Results

### 3.1. Isolation of MRSA from Clinical Samples

Of the 520 *S. aureus* isolated during the study period, 132 MRSA isolates were considered for a detailed study. Based on the criteria described in the methods section, 81 (61.4%) isolates were identified as CA-MRSA and 51 (38.6%) as HA-MRSA.

MRSA were isolated from various clinical samples such as pus, blood, a throat swab, body fluids and urine (Table 1). Of the 132 MRSA cases, 94 (71.2%) were inpatients and 38 (28.8%) were outpatients. A significant difference was observed between the isolation of CA-MSSA and HA-MSSA from the inpatient and outpatient groups (*p* < 0.05) (Table S2). The patients included in this study were in the age range of 1 to 79 years with a median age of 40 years. The gender-wise analysis of the MRSA cases revealed that 78 (59.1%) were males and 54 (40.9%) were females. No significant difference (*p* > 0.05) was observed between the incidences of CA-MRSA and HA-MRSA with regards to the age, gender, sample types or mortality rates (Table 1) (Supplementary Tables S3–S5).

### 3.2. Antibiotic Resistance Profiles of MRSA Isolates

All (100%) MRSA isolates were resistant to penicillin, cefoxitin and ampicillin (Table 2), while all MRSA isolates were susceptible to amikacin and vancomycin. CA-MRSA showed an increased susceptibility to netilmicin, linezolid, tigecycline, doxycycline, chloramphenicol, rifampicin and teicoplanin. A significant difference (*p* < 0.05) existed between CA-MRSA and HA-MRSA with regard to resistance against cephalexin, cefotaxime (β-lactam antibiotics), levofloxacin, linezolid and teicoplanin (non-β-lactam antibiotic) (Table 2). Altogether, 106 (80.3%) MRSA isolates were multidrug-resistant, of which 60 were CA-MRSA and 46 were HA-MRSA.

### 3.3. Molecular Characterization of MRSA

#### 3.3.1. SCC*mec* Typing

All (100%) of the 132 MRSA isolates harbored the *mecA* gene (Figure S1). The SCC*mec* typing results showed the presence of SCC*mec* types IV (50.6%) and V (66.7%) among CA-MRSA. HA-MRSA predominantly carried SCC*mec* types III (19.6%) and I (41.2%) (Table 3, Figures S2–S5). Further, HA-MRSA isolates harbored SCC*mec* types IV (31.4%) and V (25.5%). SCC*mec* types IV and V and *PVL* were detected together in 12.3% of CA-MRSA.

#### 3.3.2. Distribution of the lukS/F-PV Gene in MRSA

In this study, 70.4% of CA-MRSA isolates were PVL-positive based on the PCR amplification of the *lukS/F-PV* gene (Figure S6). PVL was found in 43.1% of MRSA isolates from patients with hospital-associated risk factors, which also showed the presence of SCC*mec* type IV and SCC*mec* type V. Among the true HA-MRSA, only 7.8% possessed the PVL gene.

## 4. Discussion

### 4.1. Prevalence of CA-MRSA in Clinical Specimens

This study shows the prevalence of CA-MRSA in patients without risk factors and in patients with risk factors for hospital-associated infection, as reported by previous studies [6,7]. We report a high prevalence (81 isolates, 61.4%) of CA-MRSA among the MRSA isolates of this study. A study from a rural area in Andhra Pradesh, India showed a CA-MRSA prevalence of 64.7% [34]. Other reports on CA-MRSA in India were by D’Souza et al. [11] from Mumbai, Shenoy et al. [35] from Mangalore and Bouchiat et al. from Bangalore [36]. Goud et al. reported a nasal carriage rate of 72.7% for MRSA in healthy individuals in Bangalore, India [37]. A study conducted by the Indian Network for Surveillance of Antimicrobial Resistance (INSAR) group, India from January 2008 to December 2009 in 15 tertiary care centers reported an overall MRSA prevalence of 41% [38]. A meta-analysis on CA-MRSA carriage in the Asia–Pacific region from 2000–2016 showed a 0% to 23.5% occurrence in the general public and 0.7% to 10.4% occurrence in hospital settings, and reported the highest carriage rate of 16.5% to 23.5% in India [39]. CA-MRSA prevalence varies worldwide [40,41,42]. A study from southwest Finland reported increasing CA-MRSA cases from 13% in 2007 to 43% in 2016 [43]. In Denmark, ST97-IVa MRSA clone was responsible for sporadic outbreaks in a surgical ward over a period of four years [44].

In this study, MRSA was isolated from patients with mild skin infections to severe systemic disease. The clinical details showed no significant difference (*p* > 0.05) between the different age groups and gender of patients with respect to the prevalence of CA-MRSA and HA-MRSA. CA-MRSA was almost equally isolated from outpatients (44.4%) and inpatients (55.6%), suggesting the prevalence of CA-MRSA in community and hospital settings alike, while 96.1% of HA-MRSA was from inpatients. A study by the INSAR group reported the isolation of MRSA from 43% of outpatients and 42% of inpatients [38]. In our study, no significant difference (*p* > 0.05) in the mortality rate was recorded between CA-MRSA and HA-MRSA patients.

### 4.2. Molecular Characterization of MRSA Isolates

The MRSA were further categorized by SCC*mec* typing and the presence of the PVL gene by PCR. Based on the CDC definition of no hospital-associated risk factors and the results of SCC*mec* typing, 81 isolates were classified as true CA-MRSA. These included SCC*mec* type IV (41 isolates, 50.6%), SCC*mec* type V (54 isolates, 66.7%) and PVL-positive isolates (57 isolates, 70.4%). A study from Mumbai, India reported the prevalence of SCC*mec* type IV (34.4%), SCC*mec* type V (41%) and PVL (64%) in MRSA isolates [13]. In our study, the higher prevalence of the PVL gene (70.4%) in CA-MRSA might suggest the frequent occurrence of virulent strains of CA-MRSA in inpatient and outpatient MRSA cases. Other studies from India corroborate our finding of a higher prevalence of the PVL gene in CA-MRSA. A study from North India reported PVL in 56.9% of CA-MRSA [45], while a study from Belgaum, India reported an 85.1% prevalence of PVL in MRSA [46]. In this study, the three genes SCC*mec* type IV, SCC*mec* type V and *PVL* were detected together in 12.3% of CA-MRSA. 

Of 51 HA-MRSA isolates in this study, 16(31.4%) isolates had SCC*mec* type IV, 13(25.5%) isolates had SCC*mec* type V, while the *PVL* gene was found in 22 isolates (43.1%). This is significant and might suggest the infiltration of CA-MRSA into hospital settings. Our observations are in line with a previous study from Mumbai in which 21% of MRSA strains isolated from patients with risk factors had SCC*mec* type IV or SCC*mec* type V [13]. A similar report from Chennai, India found 44.4% HA-MRSA with SCC*mec* type IV and SCC*mec* type V [47]. However, future studies employing whole genome sequencing will shed light on how CA-MRSA evolve to establish in hospital environments and cause infections.

This study suggests that virulent strains of CA-MRSA can be encountered in hospital settings and cause severe infections, which can delay the patient prognosis in the absence of a systematic diagnosis. The presence of SCC*mec* type IV and SCC*mec* type V in considerable percentages of *S. aureus* isolated from MRSA cases belonging to different medical departments is a cause for concern. The sources of CA-MRSA in the hospital environment could be the patients, MRSA carrier individuals attending the hospital or the medical staff. Of 51 HA-MRSA isolates, 25 were true HA-MRSA with SCC*mec* type I and SCC*mec* type III, and 7.8% of these harbored the PVL gene. None of the HA-MRSA isolates showed the presence of SCC*mec* type II, an observation in agreement with previous studies from India [13,20]. The presence of PVL in multidrug-resistant (MDR) HA-MRSA isolates can potentially complicate the treatment.

### 4.3. Antibiotic Resistance 

Studies have shown that CA-MRSA are more susceptible to non-β-lactam antibiotics compared to HA-MRSA [48,49]. In this study, 23 antibiotics were used to understand the antibiotic resistance profiles of MRSA isolates. CA-MRSA and HA-MRSA showed a significant (*p* < 0.05) difference in their susceptibilities to β-lactam and non-β-lactam antibiotics. Susceptibility to non-β-lactam antibiotics has been previously reported by several investigators [13,35,49]. In this study, MRSA from patients with hospital-associated risk factors and harboring the SCC*mec* type IV and SCC*mec* type V genes showed a higher antibiotic resistance similar to HA-MRSA.

In our study, CA-MRSA isolates resistant to three or more classes of antibiotics were found. These isolates were resistant to cefotaxime (44.4%), gentamicin (40.7%), ciprofloxacin (86.4%), clindamycin (40.7%), erythromycin (66.7%) and ofloxacin (49.4%). All (100%) MRSA isolates were resistant to penicillin, ampicillin, cefoxitin and oxacillin, while none showed resistance to amikacin and vancomycin. CA-MRSA was largely susceptible to netilmicin, linezolid, tigecycline, doxycycline, chloramphenicol, rifampicin and teicoplanin. A study from India reported CA-MRSA resistant to gentamicin (69%), erythromycin (62%), cotrimoxazole (58.6%) and ciprofloxacin (79.3%) [36]. MDR CA-MRSA has been reported from India and worldwide [16,17,18,34,50].

The emerging MDR resistance pattern of CA-MRSA has to be controlled with proper antibiotic stewardship.CA-MRSA isolated from patients with hospital-associated risk factors with MDR similar to HA-MRSA can lead to the spread of multidrug-resistant virulent strains of CA-MRSA in the hospital and the community. 

The molecular characterization results show an increasing trend in the prevalence of MRSA in the general population and the presence of CA-MRSA in the hospital environment as well as in patients with hospital-associated risk factors. This emphasizes that the diagnosis of CA-MRSA should not be strictly based on the risk factors but on standard diagnostic tools such as molecular characterization by PCR and antibiotic susceptibility profiles in order to avoid treatment failures.

## 5. Conclusions

This study reports the prevalence of CA-MRSA in community and hospital settings, and the study suggests the possibility of MDR CA-MRSA replacing HA-MRSA in hospitals. This needs an action plan with proper antibiotic stewardship and treatment regime in order to control the spread of CA-MRSA in hospitals and in the community. The implementation of strict aseptic techniques in hospitals to prevent the colonization of the hospital environment by resistant strains, the identification and treatment of carriers, and the screening of hospital staff and facilities are some of the key measures that can mitigate the spread of CA-MRSA.

## Figures and Tables

**Table 1:** Isolation of MRSA from various clinical samples.

Sample	CA-MRSA (*n* = 81)	HA-MRSA (*n* = 51)	Chi-Square Value	*p*-Value
Pus	72	39	7.256	0.123
Blood	07	11		
Body fluid	00	01		
Throat swab	01	00		
Urine	01	00		

**Table 2:** Antibiotic resistance profiles of MRSA.

Antibiotic Class	Antibiotics	MRSA (*n* = 132)		Chi-Square Value	*p*-Value
		CA-MRSA	HA-MRSA		
		(*n* = 81) (Resistant %)	(*n* = 51) (Resistant %)		
β–Lactam antibiotics					
Penicillins	Ampicillin	81 (100)	51 (100)	NA	NA
	Oxacillin	81 (100)	51 (100)	NA	NA
	Penicillin	81 (100)	51 (100)	NA	NA
Cephalosporins	Cephalexin	18 (22.2)	28 (54.9)	14.721	<0.001 *
	Cefoxitin	81 (100)	51 (100)	NA	NA
	Cefotaxime	36 (44.4)	36 (70.6)	8.627	0.003 *
Non-β–Lactam antibiotics					
Aminoglycosides	Amikacin	0 (0)	0 (00)	NA	NA
	Gentamycin	33 (40.7)	20 (39.2)	0.030	0.862
	Netilmicin	2 (2.5)	2 (3.9)	0.225	0.636
Fluroquinolones	Ciprofloxacin	70 (86.4)	41 (80.4)	0.850	0.357
Macrolide	Clindamycin	33 (40.7)	24 (47.1)	0.509	0.475
	Erythromycin	54 (66.7)	41 (80.1)	2.923	0.087
Sulphonamides	Trimethoprim-Sulfamethoxazole	23 (28.4)	20 (39.2)	1.668	0.196
Quinolone	Levofloxacin	10 (12.3)	21 (41.2)	14.836	0.001 *
	Ofloxacin	40 (49.4)	30 (58.8)	1.120	0.290
Oxazolidinones	Linezolid	0 (0)	6 (11.8)	9.983	0.002 *
Rifamycin	Rifampicin	9 (11.1)	8 (15.7)	0.584	0.445
Glycylcycline	Tigecycline	0 (0)	1 (2)	1.600	0.206
Tetracycline	Doxycycline	5 (6.2)	5 (9.8)	0.589	0.443
	Tetracycline	10 (12.3)	10 (19.6)	1.284	0.257
Chloramphenicol	Chloramphenicol	3 (3.7)	3 (5.9)	0.342	0.558
Glycopeptide	Teicoplanin	1 (1.2)	7 (13.7)	8.576	0.003 *
	Vancomycin	0 (0)	0 (0)	NA	NA

N/A- Not applicable, * significant.

**Table 3:** Prevalence of the SCC*mec* and PVL genes among the MRSA isolates.

Gene	N/P	MRSA (*n* = 132)			Chi-Square Value	*p*-Value
		CAMRSA (*n* = 81)	HA-MRSA (*n* = 51)			
			HA-MRSA with CA-MRSA Gene (*n* = 25)	HA-MRSA (*n* = 26)		
*mecA*	N	0	0	0	NA	NA
	P	81	25	26		
SCC*mec* type I	N	81	25	5	101.813	<0.001 *
	P	0	0	21		
SCC*mec* type II	N	81	25	26	NA	NA
	P	0	0	0		
SCC*mec* type III	N	81	25	16	44.111	<0.001 *
	P	0	0	10		
SCC*mec* type IV	N	40	9	26	26.001	<0.001 *
	P	41	16	0		
SCC*mec* type V	N	27	12	26	35.018	<0.001 *
	P	54	13	0		
PVL	N	24	3	22	33.833	<0.001 *
	P	57	22	4		

N/P- Negative/Positive, NA- Not applicable, * significant.

## Data Availability

The data presented in this study are available on request from the corresponding author.

## Supplementary Materials

The following are available online at https://www.mdpi.com/2079-6382/10/2/197/s1, Table S1: Primers used for the amplification of SCC*mec* and PVL gene, Table S2: Prevalence of MRSA among inpatients and outpatients, Table S3: Prevalence of MRSA among patients of different age, Table S4: Prevalence of MRSA among males and females, Table S5: Outcome of patients with MRSA cases; Figure S1: PCR assay for detection of *mecA* gene in MRSA isolates; Figure S2: PCR assay for detection of SCC*mec* type IV in MRSA isolates; Figure S3: PCR assay for detection of SCC*mec* type V in MRSA isolates; Figure S4: PCR assay for detection of SCC*mec* type III in MRSA isolates; Figure S5: PCR assay for detection of SCC*mec* type I in MRSA isolates; Figure S6: PCR assay for detection of PVL gene in MRSA isolates.
