# Supplementary material for: Prevalence and Characterization of Methicillin-Resistant Staphylococcus aureus from Community- and Hospital-Associated Infections: A Tertiary Care Center Study
Source: Antibiotics (Basel). 2021 Feb 18;10(2):197. doi: 10.3390/antibiotics10020197 (PMC7922968; doi:10.3390/antibiotics10020197)
Supplement: Supplementary file 1 [file antibiotics-10-00197-s001.pdf]

Table S1: Primers used for the amplification of SCC*mec* and PVL genes

Primers	Oligonucleotide sequence (5′-3′)	Size (bp)	SCC*mec* type	Reference
1.MECA P4	TCCAGATTACAACTTCACCAGG	162	*mecA*	[27,29]
2.MECAP7	CCACTTCATATCTTGTAACG			
3.CIF2F2	TTCGAGTTGCTGATGAAGAAGG	495	1	[27,29]
4.CIF2R2	ATTTACCACAAGGACTACCAGC			
5.KDPF1	AATCATCTGCCATTGGTGATGC	484	11	[27.29]
6.KDPR1	CGAATGAAGTGAAAGAAAGTGG			
7.MEC1P2	ATCAAGACTTGCATTCAGGC	209	111	[27,29]
8.MEC1P3	GCGGTTTCAATTCACTTGTC			
9.DCSF2	CATCCTATGATAGCTTGGTC	342	1V	[27,29]
10.DCSR1	CTAAATCATAGCCATGACCG			
11.TypeV-F	GAACATTGTTACTTAAATGAGCG	325	V	[28,29]
12.TypeV-R	TGAAAGTTGTACCCTTGACACC			
PVL Primers				
13.PVL-1	ATCATTAGGTAAAATGTCTGGACATGATCCA	433	PVL	[29]
14.PVL-2	GCATCAAGTGTATTGGATAGCAAAAGC			

Table S2: Prevalence of MRSA among Inpatients and Outpatients

IP/OP	CAMRSA (n=81) (%)	HAMRSA (n=51) (%)	Chi-Square value	*P* value
IP	45 (55.6)	49 (96.1)	25.068	<0.001*
OP	36 (44,4)	2 (3.9)		

IP - Inpatient (Patient requiring overnight hospitalization), OP - Outpatient (Patient visiting the hospital without hospital stay), *significant.

TableS3: Prevalence of MRSA among patients of different age

Age(years)	MRSA (n=132)		Chi-square value	*P*-value
	CA-MRSA (n=81) (%)	HA-MRSA (n=51) (%)		
>1-10	9 (11.1)	2 (3.9)		
11-20	6 (7.4)	3 (5.9)		
21-30	21 (25.9)	8 (15.7)		
31-40	12 (14.8)	8 (15.7)	8.969	0.255
41-50	13 (16)	9 (17.6)		
51-60	13 (16)	10 (19.6)		
61-70	4 (4.9)	9 (17.6)		
71-80	3 (3.7)	2 (3.9)		

Table S4: Prevalence of MRSA among males and females

Gender	MRSA (n=132)		Chi-square value	*P* value
	CAMRSA (n=81) (%)	HAMRSA (n=51) (%)		
Female	37 (45.7)	17 (33.3)	1.973	0.160
Male	44 (54.3)	34 (66.7)		

Table S5: Outcome of patients with MRSA cases

Outcome	MRSA (n=132)		Chi-square value	*P* value
	CA-MRSA (n=81) (%)	HA-MRSA (n=51) (%)		
Dead	3 (3.7)	5 (9.8)		
Recovered	78 (96.3%)	46 (90.2)	2.046	0.153

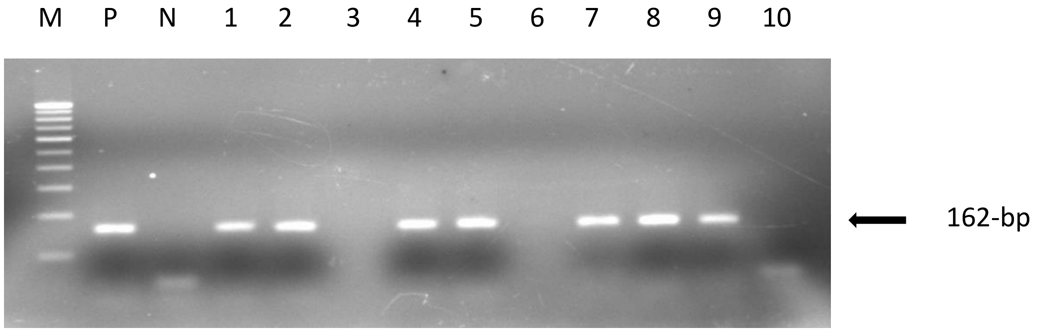

Figure 1. PCR assay for detection of *mecA* gene in MRSA isolates
Lane M: Molecular marker 100-bp DNA ladder, Lane P: Positive control,
Lane N: Negative control, Lane 1, 2, 4,5, 7, 8, 9: MRSA isolate positive for *mecA* gene
Lane 3,6 &10: MRSA isolate Negative for *mecA* gene

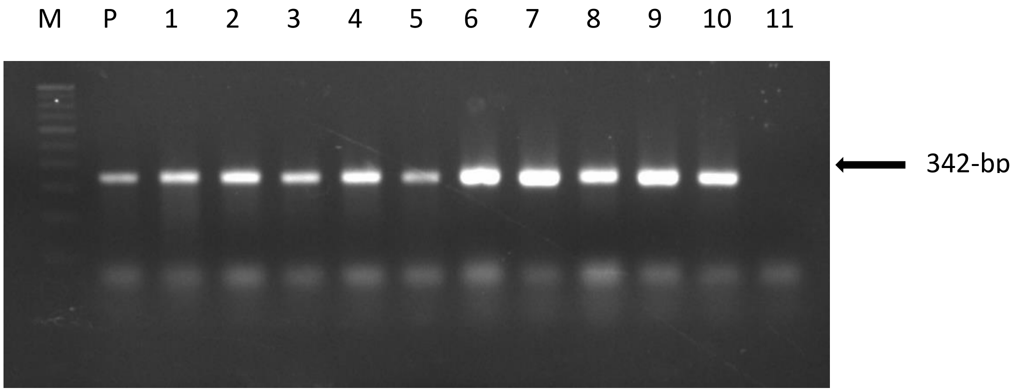

.

Figure 2. PCR assay for detection of SCC mec type IV in MRSA isolates.
Lane M: Molecular marker 100-bp DNA ladder, Lane P: Positive control,
Lane 1,2,3,4,5,6,7,8,9,10 &11: MRSA isolate positive for SCC*mec* type IV
Lane 11: Negative control .

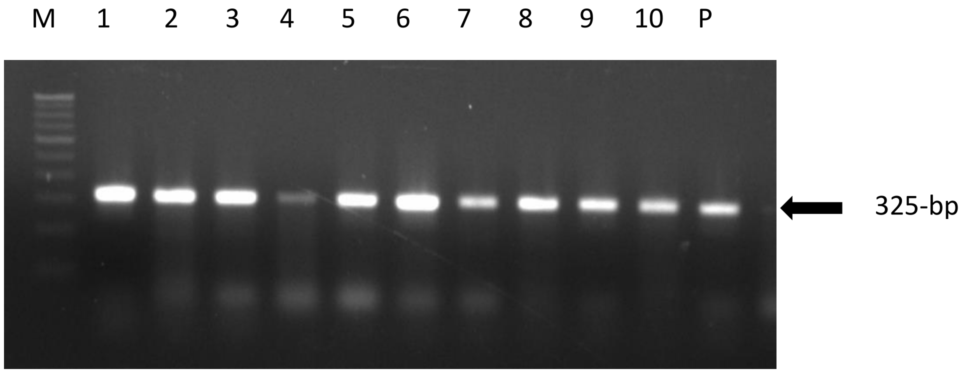

Figure 3. PCR assay for detection of SCC*mec* type V in MRSA isolates
Lane M: Molecular marker 100-bp DNA ladder,
Lane 1,2,3,4,5,6,7,8,9 &10: MRSA isolate positive for SCC*mec* type V,
Lane P: Positive control.

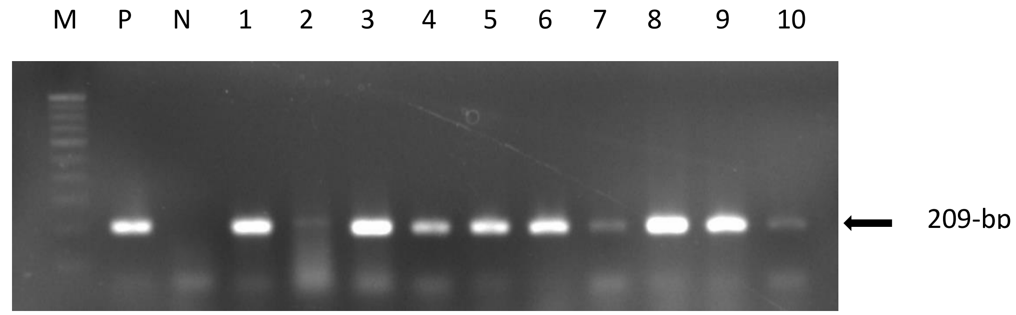

Figure 4. PCR assay for detection of SCC mec type III in MRSA isolates
Lane M: Molecular marker 100 bp DNA ladder, Lane P: Positive control,
Lane N: Negative control, Lane 1,2,3,4,5,6,7,8,9&10: MRSA isolate positive for SCC*mec* type III

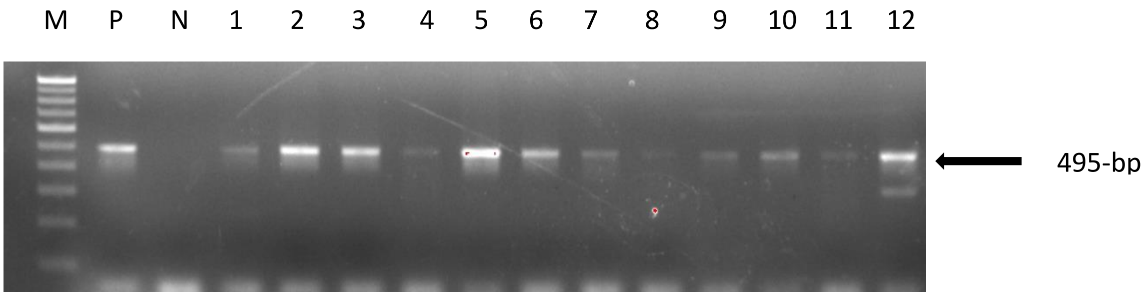

Figure 5. PCR assay for detection of SCC mec type I in MRSA isolates
Lane M: Molecular marker 100-bp DNA ladder, Lane P: Positive control,
Lane N: Negative control, Lane 1,2,3,4,5,6,7,8,910,11,&12: MRSA isolate positive for SCC*mec* type I.

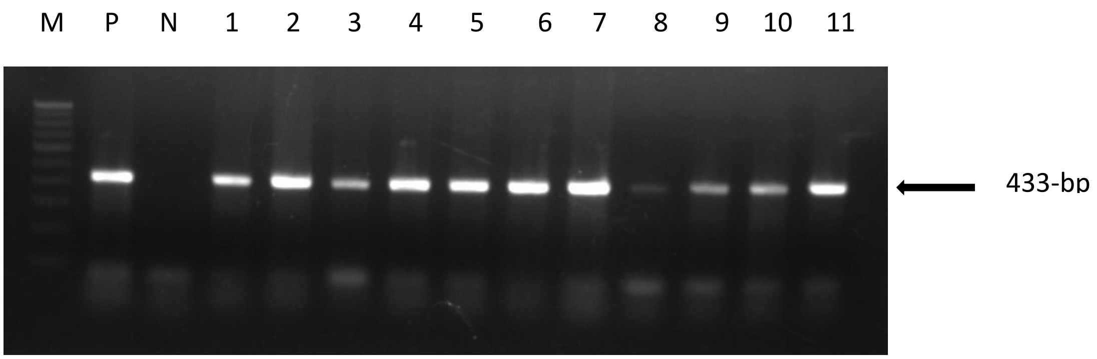

Figure 6. PCR assay for detection of PVL gene in MRSA isolates.
Lane M: Molecular marker 100-bp DNA ladder, Lane P: Positive control,
Lane N: Negative control, Lane 1,2,3,4,5,6,7,8,9,10 &11: MRSA isolate positive for *PVL* gene
.
